# miR-155-5p Implicates in the Pathogenesis of Renal Fibrosis via Targeting SOCS1 and SOCS6

**DOI:** 10.1155/2020/6263921

**Published:** 2020-06-06

**Authors:** Wanfen Zhang, Xiaoping Li, Yushang Tang, Cheng Chen, Ran Jing, Tongqiang Liu

**Affiliations:** ^1^Division of Nephrology, The Affiliated Changzhou No.2 People's Hospital of Nanjing Medical University, Changzhou, Jiangsu, China; ^2^Institute of Nephrology, Zhong Da Hospital, Southeast University, School of Medicine, Nanjing, Jiangsu, China

## Abstract

Renal fibrosis is associated with the reduction in the functional renal parenchyma and in most cases progresses to end-stage kidney failure, a devastating condition that requires lifelong dialysis or kidney transplantation. However, due to the extreme complexity in the pathogenesis of renal fibrosis and our limited knowledge, therapeutic options for renal fibrosis in the clinical setting are still scarce and often ineffective. Hence, further studies on the molecular mechanisms underlying renal fibrosis are compellingly needed. Multiple miRNAs have demonstrated to participate in kidney diseases in a TGF-*β* dependent or independent manner, but there is very little known about miR-155-5p on renal fibrosis. In the present study, we firstly explored the expression level and functions of miR-155-5p in the setting of renal fibrosis. Our research revealed that miR-155-5p is highly expressed in kidney tissues from patients and unilateral ureteral obstruction (UUO) rat models, and miR-155-5p knockdown significantly blocks renal fibrosis both *in vivo* and *in vitro*. In mechanism, our data demonstrate that miR-155-5p promotes renal fibrosis by increasing the phosphorylated activation of STAT3 via targeting SOCS1/6. Altogether, our findings highlight a miR-155-5p/SOCS/STAT3 axis in the pathogenesis of renal fibrosis, which may provide promising therapeutic targets for clinical prevention of this disease.

## 1. Introduction

Renal fibrosis, characterized by tubulointerstitial fibrosis and glomerulosclerosis, is almost the final pathological process of chronic kidney disease (CKD) and ultimately causes kidney failure and organ death in most cases [1–4]. In histological features, renal fibrosis manifests the excessive accumulation and deposition of extracellular matrix components, which is always companied by various pathological alterations in cells including epithelial-to-mesenchymal transition, fibroblast activation, immune cell infiltration, and apoptosis [1–3]. In recent epidemiological studies, in the United States, over 30 million people already have been diagnosed as CKD, and around 50% of people are projected to develop the disease in their lifetime [5]. Currently, due to the complexity in the pathogenesis of renal fibrosis, the effective intervention for this disease is still lacking. Hence, further studies on the molecular mechanisms underlying renal fibrosis are compellingly needed.

Previously, tremendous evidence has revealed that TGF-*β* is substantially upregulated in renal fibrosis, and overexpression of active TGF-*β* is capable of inducing renal fibrosis [4, 6]. In contrast, blocking TGF-*β* using a neutralizing antibody, antisense oligonucleotides, inhibitors, or genetic deletion of receptors effectively alleviates the degree of renal fibrosis both *in vivo* and *in vitro* [7]. In the progression of renal fibrosis, TGF-*β*1 signaling promotes the deposition of the extracellular matrix via activating transcriptional factors either Smad (such as Smad2/3/4) or STAT (such as STAT3) family [6]. Kuratsune and colleagues reported that a significantly elevated activation of STAT3 is observed in the renal tubular interstitial cells of the kidneys with unilateral ureteral obstruction (UUO) [8]. Moreover, Pang and colleagues demonstrated that S3I-201, a widely used STAT3 inhibitor, remarkably suppresses the expression of fibronectin, *α*-SMA (alpha-smooth muscle actin), and collagen I proteins and effectively alleviates UUO-induced renal interstitial fibrosis of animals [9]. All the previous evidence indicates that STAT3 plays a critical role in the pathogenesis of renal fibrosis. However, why the expression of STAT3 is upregulated in fibrotic kidney tissues remains unclear.

MicroRNAs (miRNAs), a kind of well-investigated small endogenous noncoding RNAs with 20-22 nucleotides in length, are ultimately assembled into the RNA-induced silencing complex (RISC) to bind the 3′UTR of target mRNAs for the posttranscriptional gene silencing by mRNA degradation or translation inhibition [10, 11]. Extensive miRNAs have been demonstrated to participate in kidney development, hemostasis, and especially kidney fibrosis in a TGF-*β*-dependent or TGF-*β*-independent manner, such as miR-21, miR-23a, miR-27, miR-34a-5p, miR-93, miR-132, miR-135, and miR-146 which are reported highly expressed in renal fibrotic tissues and contribute to the fibrogenesis of renal; while miR-23b, miR-29, miR-30, miR-34a, miR-192, miR-200, and miR-433 are described downregulated and revealed an inhibitory effect on kidney fibrosis [12–18].

miR-155-5p, a typical multifunctional miRNA, is first identified in B-cells and is processed from an exon of a noncoding RNA transcribed from the B-cell Integration Cluster (BIC) located on the chromosome [19–21]. miR-155-5p is ubiquitously expressed in various tissues and plays crucial roles both in physiological and pathological processes including hematopoietic lineage differentiation, immunity, inflammation, cancer, and cardiovascular diseases [21–23]. In the kidney, miR-155-5p was reported abnormally expressed in renal cancer, end-stage renal disease, and renal transplantation undergoing acute cellular rejection [24–30]. More importantly, miR-155-5p was observed to be upregulated in fibrotic lung tissues, and hypoxia-induced miRNA-155-5p promotes the fibrosis of proximal tubule cells *in vitro* [31–35], implying that it may participate in the pathology of renal fibrosis. However, whether and how miR-155-5p involves renal fibrosis remain unknown.

In this study, we firstly explored the expression level and functions of miR-155-5p in renal fibrosis patients and unilateral ureteral obstruction (UUO) rat models. Noticeably, due to the capability of generating progressive renal fibrosis, UUO has become one of the most popular models for chronic kidney disease research *in vivo* [36]. We observed that miR-155-5p was highly expressed in the kidney tissues from renal disease patients and UUO rats. To further investigate the underlying mechanisms by which miR-155-5p involved in the pathogenesis of renal fibrosis, we identified SOCS1/6 as its putative targets using bioinformatics tools, and these results were experimentally validated in NRK-49F and HK-2 cells and clinical specimens. Finally, our work revealed that miR-155-5p promotes renal fibrosis by increasing the phosphorylated activation of STAT3 both *in vitro* and *in vivo.* Altogether, our findings highlight a miR-155-5p/SOCS/STAT3 axis in the pathogenesis of renal fibrosis, which may provide promising therapeutic targets for clinical prevention or treatment of this disease.

## 2. Materials and Methods

### 2.1. Patients

In this study, 6 patients (4 males and 2 females; mean age = 51.3 ± 4.7 years old) with tubulointerstitial fibrosis (TIF) disease were collected from the Affiliated Changzhou No.2 People's Hospital of Nanjing Medical University. Six healthy donors (ND; 3 males and 3 females; mean age = 49.6 ± 10.1 years old) who died from traffic accidents were enrolled as normal controls with the following exclusion criteria: with other renal diseases, 20 years of age, inability to provide informed consent, presence of active infection, and pregnancy. The kidney tissue was harvested, and RNA samples and protein samples were extracted using the Qiagen RNeasy Mini Kit and RIPA buffer, respectively. All experimental protocols and procedures involved in the present study were approved by the Ethics Committee of the Southeast University, and the study complied with the declaration of Helsinki. The written informed consent was obtained from all participants for the publication of this article.

### 2.2. Unilateral Ureteral Obstruction (UUO) Rat Models

All animal experiments were performed following the protocols approved by the Southeast University institutional committee for the care and use of laboratory animals and compliance with local authorities (2018-2404). Adult male rats (250-300 g body weight) were purchased from Shanghai SLAC Laboratory Animal Co., Ltd. (Shanghai, China). All animals were housed in sterile and filter-capped cages in the present study. The surgery of UUO was carried out as previously described [37]. In brief, animals were anesthetized and a midline incision was made in the abdominal wall, the left ureter was dissected out and ligated with 4.0 silk at two points along its length, and the kidneys were harvested at the seventh day after surgery.

### 2.3. Cell Culture, Transfection, and miR-155-5p Knockdown

HEK293 cell line and kidney cell lines NRK-49F and HK-2 were maintained in DMEM medium plus with 10% heat-inactivated fetal bovine serum and 1% Penicillin-Streptomycin and kept in culture with 37°C and 5% CO_2_ condition. For plasmid transfection, the cells were seeded in the 6-well plates and transfected with Lipofectamine 2000 (Invitrogen, Carlsbad, CA, USA) following the instructions. For miR-155-5p knockdown, shRNA-expressing lentivirus was packaged in HEK293 cells by cotransfected vectors and packaging plasmids; 48 hours later, the virus was harvested and subjected to infect cells. The miR-155-5p knockdown cells were obtained by selecting with puromycin.

### 2.4. Luciferase Reporter Assay

HEK293 cells were transfected with the indicated reporter constructs containing either the wild-type or mutated miR-155-5p binding sequences in the 3′UTRs of SOCS1, SOCS5, and SOCS6 and control Renilla luciferase reporter and miR-155-5p or NC. Luciferase activity was determined by using a dual luciferase assay kit (Promega, Madison, WI, USA) 48 hours after transfection, and firefly luciferase activity was measured and normalized with cotransfected Renilla luciferase activity.

### 2.5. RNA Extraction and Real-Time Quantitative Polymerase Chain Reaction (RT-qPCR)

Total RNA was extracted from human renal disease patients, rat kidney tissues, or cultured cells by TRIzol reagent or Qiagen RNeasy Mini Kit according to the manufacturer's instruction. An equal amount RNA was subjected to reverse the transcription for cDNAs using the Takara PrimeScript RT-PCR Kit (Takara, Dalian, China). Real-time PCR was carried out using the TB Green Fast qPCR Mix (Takara, Dalian, China). Quantification was done using *^ΔΔ^*Ct values, and the reference gene GAPDH was used as the internal control.

For the detection of mature miR-155-5p, the One-Step PrimeScript miRNA cDNA Synthesis Kit (Takara, Dalian, China) was used for RNA reverse transcription. The relative expression of miRNA was calculated using the Ct method, and the expression of U6 small nuclear RNA (snRNA) was used as an internal control. The primers used in the study were listed as follows: SOCS1 forward: 5′-CAC GCA CTT CCG CAC ATT C-3′; SOCS1 reverse: 5′-TAA GGG CGA AAA AGC AGT TCC-3′; SOCS5 forward: 5′-GTG CCA CAG AAA TCC CTC AAA-3′; SOCS5 reverse: 5′-TCT CTT CGT GCA AGT CTT GTT C-3′; SOCS6 forward: 5′-ATC ACG GAG CTA TTG TCT GGA-3′; SOCS6 reverse: 5′-CTG ACT CTC ATC CTC GGG GA-3′; GAPDH forward: 5′-ATC ACT GCC ACC CAG AAG AC-3′; GAPDH reverse: 5′-ATG AGG TCC ACC ACC CTG TT-3′; miR-155-5p forward: 5′-CTT AAT GCT AAT CGT GAT AGG GGT-3′; U6 forward: 5′-GCT TCG GCA GCA CAT ATA CTA AAA T-3′; and U6 reverse: 5′-CGC TTC ACG AAT TTG CGT GTC AT-3′. Noticeably, the reverse primer of miR-155-5p is the universal primer from the company.

### 2.6. Immunoblotting (IB)

Cells or kidney tissues were lysed in RIPA buffer and quantitated using a BCA kit (Abcam, Cambridge, MA, USA). Subsequently, the protein samples were separated by SDA-PAGE and then transferred to PVDF (polyvinylidene difluoride) membranes (Millipore, Billerica, MA, USA). Then, the membranes were blocked with 5% BSA and blotted with primary antibodies overnight. The next day, the immunoreactive bands were exposed using the enhanced chemiluminescence (ECL). *β*-Actin (or *β*-tubulin) was used as internal control. Antibodies against human SOCS1 (#3950), STAT3 (#12640), phospho-STAT3 (Tyr705) (#9145), *α*-smooth muscle actin (#19245), *β*-tubulin (#2128) were purchased from Cell Signaling Technology (Cell Signaling Technology, USA); antibodies of SOCS5 (ab97283), SOCS6 (ab197335), fibronectin (ab2413), collagen I (ab34710), and *β*-actin (ab8227) were purchased from Abcam (Abcam, Cambridge, MA, USA).

### 2.7. Hematoxylin and Eosin (H&E) Staining and Immunohistochemistry (IHC)

For histological analysis, rats or renal disease patient's kidney tissues were collected and embedded in paraffin, and 4 *μ*m tissue sections were subjected to hematoxylin and eosin staining. The interstitial injury was scored in a blinded manner according to the widely used semiquantitative scale for renal tubular injury: no injury (0), mild: less than 25% (1), moderate: less than 50% (2), severe: less than 75%, and (3) very severe: more than 75% (4). Immunohistochemical analysis was applied in paraffin sections that were deparaffinized in xylene and rehydrated in a gradient of ethanol and distilled water. Antigen retrieval was finished with a microwave-based technique. Tissue sections were then blocked with 5% BSA and subjected to primary antibodies collagen I and *α*-SMA. Isotype-matched rabbit immunoglobulin G was used as negative controls, and the nuclei were counterstained with hematoxylin.

### 2.8. *In Situ* Hybridization (ISH)

For *in situ* hybridizations, specific 5′FITC-labeled antisense-locked nucleic acid oligonucleotides for mmu-miR-155 and a scrambled probe as negative control were obtained commercially. Paraffin-embedded kidney tissue slides were deparaffinization and deproteinization and subsequently were prehybridized with hybridization buffer. The hybridization was carried out overnight with the FITC-anti-sense microRNA probe at 45°C. Signals were visualized and detected under the fluorescence microscope.

### 2.9. Bioinformatics Analysis and Statistics Analysis

Potential miR-155-5p targets were predicted with in-house bioinformatics tools MicroCible and MicroTopTable. In this study, all the data were expressed as mean ± SEM, and the duplication numbers were specified in the figure legends. Statistical analysis was carried out using the software of GraphPad Prism7 (San Diego, CA, USA). For the comparison of two samples, the student's *t*-test was used after *F*-test to compare their variances; for the comparison of multiple samples, the one-way ANOVA test was used; in the linear regression for correlation analysis, *R*^2^ was calculated for the fit goodness. *p* value < 0.05 was considered statistically significant.

## 3. Results

### 3.1. miR-155-5p Is Highly Expressed in the Fibrotic Kidney Tissues

**Figure 1:**
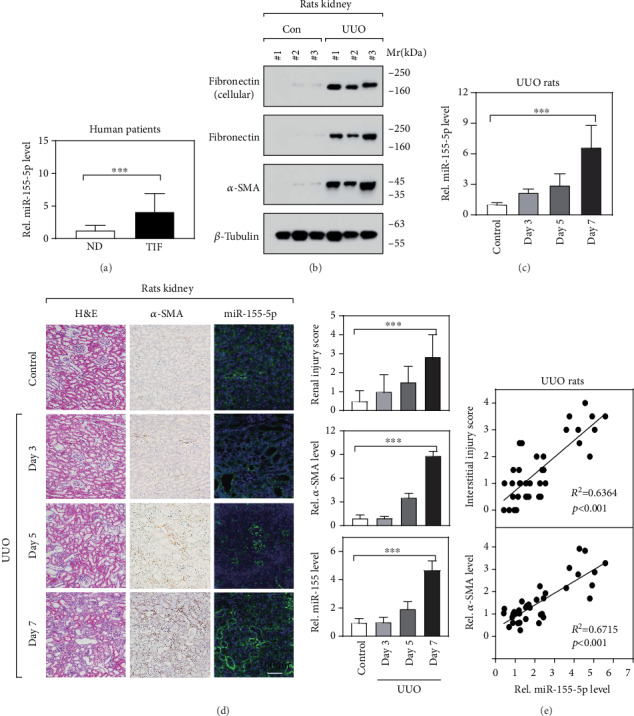
miR-155-5p is highly expressed in the fibrotic kidney tissues. (a) RT-qPCR showed the expression level of miR-155-5p in the kidney tissues from normal donors (ND, *n* = 6) and tubulointerstitial fibrosis (TIF) patients (*n* = 6). ^∗∗∗^*p* < 0.001 by *t*-test. (b) Immunoblot determined the protein levels of fibronectin and *α*-SMA in the kidney tissues. UUO: unilateral ureteral obstruction surgery; Mr: molecular weight; KDa: kilo Dalton. (c) RT-qPCR determined miR-155-5p expression in the kidney from UUO rats at different time points (after UUO surgery). *n* = 12, ^∗∗∗^*p* < 0.001 by *t*-test. (d) Histological analysis determined renal fibrosis injury (H&E staining), *α*-SMA (IHC), and miR-155-5p (ISH) expression in kidney sections. The optical density value of IHC and ISH was obtained using the ImageJ software, and all the values were normalized by the control group. *n* = 6, ^∗∗∗^*p* < 0.001 by one-way ANOVA. (e) The correlation between miR-155-5p expression and renal injury score/*α*-SMA expression in UUO rats. R: Pearson's correlation coefficient, ^∗∗∗^*p* < 0.001.

To investigate the roles of miR-155-5p in renal fibrosis, we first determined the expression level of miR-155-5p in the kidney tissues from the patients diagnosed as tubulointerstitial fibrosis (TIF). Intriguingly, we observed that, compared to normal donors, the miR-155-5p expression was significantly increased in the kidney tissues from patients (Figure 1(a)). To further confirm our observation, we generated a rat renal fibrosis model using UUO surgery, a widely used animal model for chronic kidney disease. Seven days after UUO surgery, we observed that the expressions of fibronectin and *α*-SMA were dramatically increased determined by IB (Figure 1(b)), indicating the animal models were built successfully. Consistent with the results of human patients, we also found that the miR-155-5p expression was dramatically upregulated (days 5-7) in the kidney tissues of UUO rets and highly correlated to the surgery course (Figure 1(c)). Furthermore, we visualized the expression pattern and the altered level of miR-155-5p in kidney sections using *in situ* hybridization. Our results revealed that the miR-155-5p was mainly expressed in renal tubular epithelial cells and the expression level gradually increased with the time course and *α*-SMA expression (Figures 1(d) and 1(e)). Besides, we observed that the miR-155-5p expression level was tightly correlated to the interstitial injury score of UUO rats (Figure 1(e)). Altogether, our observations indicated that miR-155-5p expression is increased in the fibrotic kidney tissues both from human patients and UUO animal models.

### 3.2. miR-155-5p Is Essential for Renal Fibrosis

**Figure 2:**
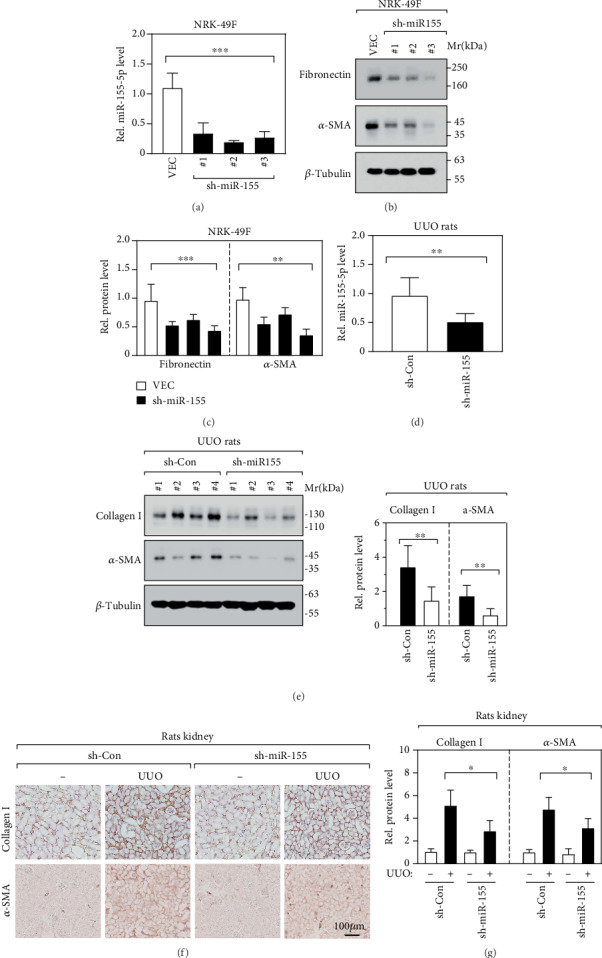
miR-155-5p is essential for renal fibrosis. (a) RT-qPCR determined miR-155-5p expression level in NRK-48F cell lines. VEC: empty vector. *n* = 6, ^∗∗∗^*p* < 0.001 by one-way ANOVA. (b, c) Fibronectin and *α*-SMA protein levels were determined by immunoblot in NRK-48F cell lines (b). The relative quantification of protein bands was carried out (c). *n* = 6, ^∗∗^*p* < 0.01 and ^∗∗∗^*p* < 0.001 by one-way ANOVA. (d) RT-qPCR revealed the miR-155-5p expression level in UUO rat renal tissues. *n* = 6, ^∗∗^*p* < 0.01 by *t*-test. (e) Immunoblot and its relative quantification represent the expression of collagen I and *α*-SMA in kidney tissues. *n* = 6, ^∗∗^*p* < 0.01 by *t*-test. (f, g) IHC (f) and its relative quantification (g) represent the expression of collagen I and *α*-SMA in kidney tissue sections. *n* = 6, ^∗^*p* < 0.05 by *t*-test.

To further explore the roles of miR-155-5p in the renal fibrogenesis, we constructed three miR-155-5p knockdown cell lines in NRK-49F using the lentivirus-mediated method. As shown in Figure 2(a), miR-155-5p expression was effectively suppressed in these NRK-49F cell lines, determined by RT-qPCR. Intriguingly, the reduction in the expression of fibronectin and *α*-SMA was observed in miR-155-5p knockdown cell lines (Figures 2(b) and 2(c)), suggesting that the knockdown of miR-155-5p alleviates renal fibrosis. Furthermore, we silenced miR-155-5p in UUO rat renal tissues using an adeno-associated virus type 2- (AAV2-) mediated shRNA-expressing system. The RT-qPCR results showed that more than 50% of miR-155-5p expression was inhibited when compared to the control shRNA-expressing tissues (Figure 2(d)). In miR-155-5p-silenced kidney tissues, a significant decrease in collagen I and *α*-SMA protein level was also observed (Figure 2(e)), indicating that the degree of renal fibrosis is alleviated. Consistently, immunostaining results also showed a dramatic downregulation of collagen I and *α*-SMA on protein level (Figures 2(f) and 2(g)). Collectively, our data indicate an essential function of miR-155-5p on renal fibrosis both *in vitro* and *in vivo*.

### 3.3. SOCS1/6 Is the Direct Targets of miR-155-5p

**Figure 3:**
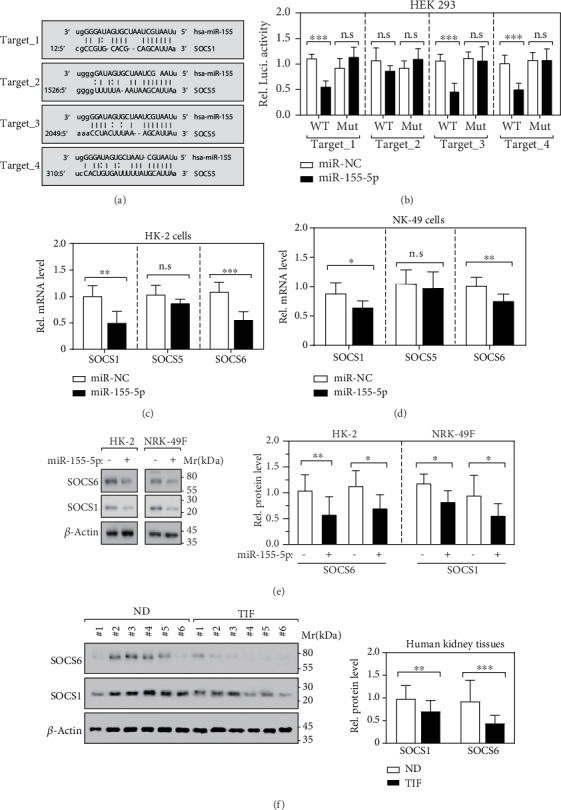
SOCS1/6 is the direct target of miR-155-5p. (a) SOCS1/5/6 are potential miR-155-5p targets predicted by in-house bioinformatics tools MicroCible and MicroTopTable. (b) Luciferase reporter assay determined the direct binding between SOCS1/5/6 and miR-155-5p, respectively. *n* = 6; n.s.: no significance, ^∗∗∗^*p* < 0.001 by *t*-test. (c) RT-qPCR determined the expression of SOCS1/5/6 in miR-155-5p overexpressed HK-2 cells. *n* = 6; n.s.: no significance, ^∗∗^*p* < 0.01 and ^∗∗∗^*p* < 0.001 by *t*-test. (d) RT-qPCR determined the expression of SOCS1/5/6 in miR-155-5p overexpressed NRK-49 cells. *n* = 6; n.s.: no significance, ^∗^*p* < 0.05 and ^∗∗^*p* < 0.01 by *t*-test. (e) Immunoblot and its relative quantification represent the expression of collagen I and *α*-SMA in HK-2 and NRK-49 cells. *n* = 3; ^∗^*p* < 0.05 and ^∗∗^*p* < 0.01 by *t*-test. (f) Immunoblot and its relative quantification represent the expression of SOCS1/6 in human kidney tissues. *n* = 12; ^∗∗^*p* < 0.01 and ^∗∗∗^*p* < 0.001 by *t*-test.

To determine the exact mechanism by which miR-155-5p is implicated in renal fibrosis, we used the bioinformatics method to predict the potential binding sequences in target genes. We found that genes SOCS1/5/6 were potentially the direct targets of miR-155-5p, indicated by the complementary sequences between their mRNA and miR-155-5p (Figure 3(a)). Subsequently, we designed luciferase reporter constructs containing either the wild-type or mutated miR-155-5p binding sequences in SOCS1/5/6 to experimentally validate the bindings with miR-155-5p. Intriguingly, we observed that target 1 (in SOCS1), target 3 (in SOCS5), and target 4 (in SOCS6) were specifically bonded by miR-155-5p (Figure 3(b)). Furthermore, we found that ectopic miR-155-5p remarkably downregulated the mRNA level of SOCS1/6 expression but not SOCS5 in HK-2 and NRK-49 cells (Figures 3(c) and 3(d)), suggesting that only SOCS1/6 is a direct target of miR-155-5p. Meanwhile, the ectopic miR-155-5p also significantly reduced the protein level of SOCS1 and SOCS6 in HK-2 and NRK-49 cells (Figure 3(e)). Besides, we determined the expression of SOCS1 and SOCS6 in TIF patients and observed a significant decrease in SOCS1 and SOCS6 protein levels when compared to ND patients (Figure 3(f)). Altogether, miR-155-5p directly targets the mRNA of SOCS1/6 both *in vitro* and *in vivo*.

### 3.4. miR-155-5p Is Involved in Renal Fibrosis via Regulating the Phosphorylation of STAT3

**Figure 4:**
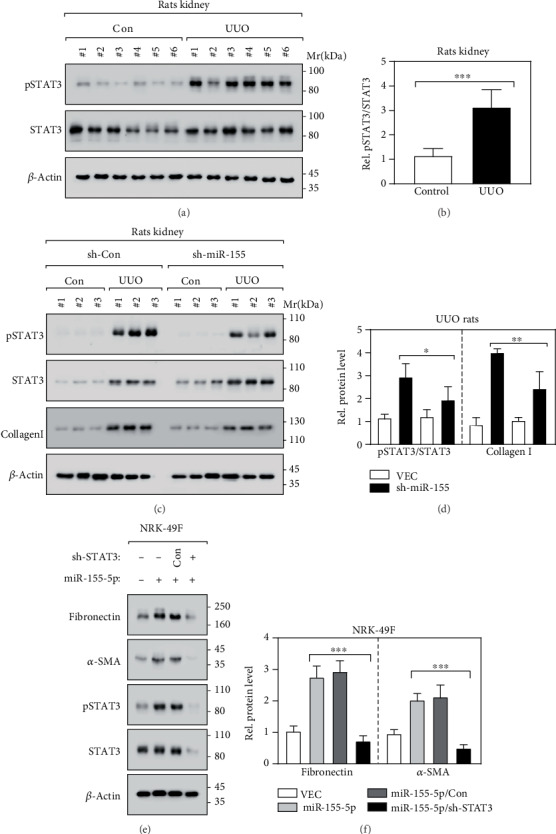
miR-155-5p is involved in renal fibrosis via regulating the phosphorylation of STAT3 A-B. Immunoblot (a) and its relative quantification (b) revealed the STAT3 phosphorylation at Y705 in kidney tissues. *n* = 12; ^∗∗∗^*p* < 0.001 by *t*-test. (c, d) Immunoblot (c) and its relative quantification (d) revealed the indicated protein levels in kidney tissues. *n* = 9; ^∗^*p* < 0.05 and ^∗∗^*p* < 0.01 by *t*-test. (e, f) Immunoblot (e) and its relative quantification (f) revealed the indicated protein levels in NRK-49 cells. *n* = 6; ^∗∗∗^*p* < 0.01 by *t*-test.

Previously, extensive studies have demonstrated that SOCS proteins are involved in the inflammatory response in the progress of renal fibrosis by negatively regulating the JAK-STAT signal cascade [38–40]. Mechanistically, SOCS proteins suppress the JAK tyrosine kinase activity via their kinase inhibitory regions (KIR) and then inhibit STAT protein phosphorylation and activation [40]. In our experiments, we observed that miR-155-5p directly targets SOCS1/6 to regulate their protein level both *in vitro* and *in vivo*. Hence, we speculated that miR-155-5p may influence the phosphorylation level of STAT3 at Y705. Indeed, we observed dramatic elevation in the Y705 phosphorylation level of STAT3 in kidney tissues of UUO rats (Figures 4(a)–4(d)). More importantly, knockdown of miR-155-5p expression in the kidney significantly alleviated the increase of STAT3 phosphorylation induced by UUO surgery (Figures 4(c) and 4(d)). These observations support our speculation mentioned above. Meanwhile, we also found that the increased protein level of collagen I induced by UUO surgery was partially reversed by knockdown miR-155-5p (Figures 4(c) and 4(d)), implying that STAT3 may mediate renal fibrosis. Therefore, we knocked down STAT3 expression in NRK-49F cells to test whether it is required for miR-155-5p's biological functions *in vitro*. Our results revealed that the elevated expression of fibronectin and *α*-SMA induced by ectopic expression of miR-155-5p in NRK-49F cells was effectively eliminated by loss function of STAT3 (Figures 4(e) and 4(f)), suggesting that miR-155-5p is involved in renal fibrosis via regulating the phosphorylation of STAT3.

## 4. Discussion

The pathogenesis of renal fibrosis is a progressive process that ultimately leads to end-stage renal failure, which is regulated by various signaling including miRNAs [1–3]. Multiple miRNAs were observed functioning on renal fibrosis through regulating numerous cellular activities including epithelial-mesenchymal transition (EMT), fibroblast activation, inflammatory reaction, and apoptosis [12–15, 28–30, 41–43]. For instance, studies have demonstrated that TGF-*β*/Smad3 promotes renal fibrosis by inducing the expression of miR-21, miR-433, and miR-192, which in turn suppressed PTEN, Smad7, ZEB1/2, and AZIN1, respectively [12–14, 16, 30, 41, 44]. Besides, TGF-*β*/Smad3 is also revealed to suppress HDAC4 and E-Cadherin expression to accelerate renal fibrosis by miR-29 and miR-200, respectively [17, 43]. Furthermore, miR-192, miR-433, and miR-21 were reported playing a pathological role in renal fibrosis through a feed-forward loop to amplify TGF-*β* signaling [14]. As a typical multifunctional miRNA, miR-155-5p is reported widely expressed in various tissues and plays a crucial role in various physiological and pathological processes [21–23]. In the kidney, miR-155-5p was reported abnormally expressed in renal cancer, end-stage renal disease, and renal transplantation undergoing acute cellular rejection [24–30], suggesting the essential effects of miR-155-5p on renal pathology. Various studies implied the tumor-promoting function of miR-155-5p on renal cancer [22, 26, 27, 45]. It is reported that miR-155-5p silencing inhibits proliferation and migration by upregulating BACH1 in renal cancer cells [46]. Besides, miR-155-5p is revealed capable of increasing the proliferation and invasion of renal cancer cells by targeting E2F2 [26]. More importantly, it is suggested that miR-155-5p may implicate in regulating renal fibrosis. For instance, in 2015, Shenping Xie and colleagues found that in HK-2 cells, HIF-1*α*-induced miR-155-5p promoted the fibrosis of proximal tubule cells by regulating both TGF-*β*1 and the process of EMT under hypoxia [35]. In the present study, we firstly explored the expression level and functions of miR-155-5p in renal fibrosis patients and unilateral ureteral obstruction (UUO) rat models. Our work demonstrated the profibrotic function of miR-155-5p on renal fibrosis *in vitro* and UUO rat models. We observed that miR-155-5p was highly expressed in the kidney tissues from renal disease patients and UUO rats. Further study revealed that knockdown miR-155-5p can significantly block renal fibrogenesis *in vitro* and *in vivo*. Our work again highlights the significance of miR-155-5p on renal fibrosis.

SOCS1 and SOCS6 are members of SOCS (suppressor of cytokine signaling) family proteins that includes 8 members, SOCS1-7 and CIS, which negatively regulate JAK/STAT pathway activation by suppressing JAK and STAT phosphorylation and affect multiple cellular activations that respond to LIF, TPO, IL-6, etc. [40]. The JAK/STAT pathway was determined as crucial mediators in renal fibrosis, via regulating EMT, apoptosis, and inflammatory response [47]. STAT3 activation was observed in rat tubular epithelial cells and myofibroblasts after UUO treatment [8], and STAT3 inhibitor S3I-201 significantly attenuates renal interstitial fibroblast activation and interstitial fibrosis [9], suggested as the profibrotic role of JAK/STAT in renal injury and fibrosis. But, early studies did not clarify the that the mechanism underlying JAK/STAT activation implicates in kidney dysfunction. Consistent with other studies, we observed a significant increase in the STAT3 activation in renal fibrosis, and miR-155-5p is capable of activating STAT3 both *in vitro* and *in vivo*. Our work showed that UUO treatment dramatically increases STAT3 phosphorylation, which was remarkably restored by miR-155-5p knocking down. Furthermore, renal fibrosis induced by miR-155-5p overexpression is blocked by STAT3 deficiency. Collectively, these data demonstrated that miR-155-5p regulates STAT3 activation and STAT3 mediated the profibrotic function of miR-155-5p on renal injury. In consideration of miR-155-5p directly targeting SOCS1 and SOCS6, there are logical reasons to assume that the molecular mechanism by which miR-155-5p regulates STAT3 phosphorylation is via targeting SOCS1 and SOCS6.

In conclusion, our work uncovered a profibrotic effect of miR-155-5p on renal injury and illustrated a potential mechanism that miR-155-5p regulating STAT3 activation by directly targeting SOCS1 and SOCS6. Our findings may provide a deep insight into renal fibrosis and a new therapeutic target for clinical prevention and treatment of renal fibrosis.

## Data Availability

The data used to support the findings of this study are available from the corresponding author upon request.

## Supplementary Materials

Supplementary Materials The statistic test results of Figure 3, indicating the significance of each panel.
